# Mapping pathological changes in brain structure by combining T1- and T2-weighted MR imaging data

**DOI:** 10.1007/s00234-015-1550-4

**Published:** 2015-06-24

**Authors:** Marco Ganzetti, Nicole Wenderoth, Dante Mantini

**Affiliations:** Neural Control of Movement Laboratory, Department of Health Sciences and Technology, ETH Zurich, Winterthurerstrasse 190, 8057 Zurich, Switzerland; Department of Experimental Psychology, University of Oxford, 9 South Parks Road, Oxford, OX1 3UD UK; Laboratory of Movement Control and Neuroplasticity, Faculty of Kinesiology and Rehabilitation Sciences, KU Leuven, Tervuursevest 101, Leuven, 3001 Belgium

**Keywords:** Brain mapping, Screening, Structural alterations, Schizophrenia, Magnetic resonance imaging

## Abstract

**Introduction:**

A workflow based on the ratio between standardized T1-weighted (T1-w) and T2-weighted (T2-w) MR images has been proposed as a new tool to study brain structure. This approach was previously used to map structural properties in the healthy brain. Here, we evaluate whether the T1-w/T2-w approach can support the assessment of structural impairments in the diseased brain. We use schizophrenia data to demonstrate the potential clinical utility of the technique.

**Methods:**

We analyzed T1-w and T2-w images of 36 schizophrenic patients and 35 age-matched controls. These were collected for the Function Biomedical Informatics Research Network (fBIRN) collaborative project, which had an IRB approval and followed the HIPAA guidelines. We computed T1-w/T2-w images for each individual and compared intensities in schizophrenic and control groups on a voxel-wise basis, as well as in regions of interest (ROIs).

**Results:**

Our results revealed that the T1-w/T2-w image permits to discriminate brain regions showing group-level differences between patients and controls with greater accuracy than conventional T1-w and T2-w images. Both the ROIs and the voxel-wise analysis showed globally reduced gray and white matter values in patients compared to controls. Significantly reduced values were found in regions such as insula, primary auditory cortex, hippocampus, inferior longitudinal fasciculus, and inferior fronto-occipital fasciculus.

**Conclusion:**

Our findings were consistent with previous meta-analyses in schizophrenia corroborating the hypothesis of a potential “disconnection” syndrome in conjunction with structural alterations in local gray matter regions. Overall, our study suggested that the T1-w/T2-w technique permits to reliably map structural differences between the brains of patients and healthy individuals.

## Introduction

The non-invasive detection of altered brain anomalies in neurological and psychiatric patients largely relies on magnetic resonance imaging (MRI) data. In particular, images from conventional T1-weighted (T1-w) and T2-weighted (T2-w) MRI, being characterized by a high contrast and spatial resolution, have been widely used to investigate structural properties of the brain [1–4]. Since being introduced into clinics several decades ago, the interpretation of T1-w and T2-w images is presently a routine task for the majority of clinicians. Importantly, the two images differ with respect to their intrinsic sensitivities towards biophysical properties of brain tissues. The T1-w image is thus more weighted towards the predisposition of a tissue to absorb energy into its lattice whereas the T2-w image is more influenced by spin-spin interaction processes [5]. This implies that, in the case of brain pathologies, structural alterations resulting from edema, inflammation, tumor infiltration, iron accumulation, or atrophy will manifest themselves differently in the two kinds of images. However, T1-w and T2-w images also have some common limitations, which primarily relate to the following: 1) the presence of spatial inhomogeneities in image intensity, produced by interactions between the subject’s body and the MR scanner and 2) the lack of image intensity standardization, mainly related to the MR instrumentation and acquisition sequence used. These factors limit the use of conventional structural MR images for the study of pathological changes in brain structure.

Nowadays, clinicians most often tend to infer brain tissue integrity through visual comparison of T1-w and T2-w images, when both are available. Also, a number of clinical studies have combined the information from the two kinds of images to study, for instance, multiple sclerosis [6] or mesial temporal lobe epilepsy [7]. The parallel analysis of T1-w and T2-w images represents the first attempt to implement a multi-modal structural imaging approach for the study of brain tissue abnormalities. However, a direct fusion of the two kinds of images, so as to produce a multi-modal T1-w/T2-w image [8, 9], could prove to be an interesting alternative. In particular, T1-w sequences are characterized by better contrast-to-noise ratio in white matter regions, whereas T2-w sequences can be used to better discriminate structural differences in fluid-filled regions [7]. Therefore, the advantages of each of these MRI techniques should be taken into account when designing clinical investigations focused on an unknown spectrum of brain pathologies. Furthermore, the comparison of different imaging modalities is advisable when no clear hypothesis can be made about which brain structures is likely to be altered.

In this study, we apply the T1-w/T2-w analysis workflow [8] to structural MRI data collected in individuals with schizophrenia and age-matched healthy controls and then compare the resulting images between groups to verify whether the technique can detect disease-related structural impairment. Since T1-w and T2-w images are most often collected in clinical scanning protocols and do not require long acquisition times, the T1-w/T2-w technique may become a widely used tool for mapping potential pathological changes resulting from brain disease.

## Methods

### Structural MR data

To validate the T1-w/T2-w approach for the detection of structural abnormalities in patients, we used MR data obtained from the Function Biomedical Informatics Research Network (fBIRN) Phase II dataset (http://fbirnbdr.birncommunity.org:8080/BDR). This was contributed by a consortium of brain imaging centers in the USA to explore the full potential of a multi-site neuroimaging approach to elucidate clinical and cognitive abnormalities in schizophrenia. The dataset includes structural and functional MR imaging (fMRI) data, as well as behavioral data, demographic information, and clinical assessments, collected in large samples of schizophrenia patients and age-matched healthy controls. Patients with schizophrenia or schizoaffective disorder were diagnosed based on the clinical interview of the Diagnostic and Statistical Manual of Mental Disorders, fourth version revised (DSM-IV-TR). Control subjects were excluded if they had a current or past history of a major neurological, psychiatric, medical illness, previous head injury, substance or alcohol dependence, or an IQ below 75. MR imaging data included structural T1-w and T2-w scans, two cognitive fMRI tasks (auditory oddball and serial item recognition tasks), two calibration fMRI scans (breath hold and sensory motor tasks), and a resting state fMRI scan. Informed consent was obtained from the participants in this study, which had itself received ethical approval from the relevant institutional review board (IRB). In the current study, we retrospectively evaluated T1-w and T2-w images from 36 patients (10 female, 40.4 ± 11.8 years old) and 35 controls (14 female, 39.1 ± 12.9 years old), coming from sites coded as 0006, 0010, and 0018. MR data from other sites were not included as they either did not contain both T1-w and T2-w images for the same subject or the scan did not cover the whole brain. MR data extracted from the sites 0006 and 0018 were collected with a 3T Siemens Trio, whereas the data extracted from the site 0010 were collected with a 1.5T Siemens Trio scanner. MR images were acquired in both patients and controls with the same scanner, using the same scanning parameters. T1-w scans were acquired in the sagittal plane using a magnetization-prepared rapid gradient echo (MPRAGE) sequence. The scanning parameters were as follows: 256 × 256 matrix, 24 cm field of view (FOV), 160–170 slices, and slice thickness 1.2–1.5 mm. T2-w scans were acquired using a turbo spin echo (TSE) sequence in the oblique axial plan tilted to be parallel to the AC-PC plane. The scanning parameters were as follows: 256 × 192 matrix, 22 cm FOV, 27 slices, and slice thickness 4 mm with a 1-mm gap.

### Imaging analysis

**Fig. 1:**
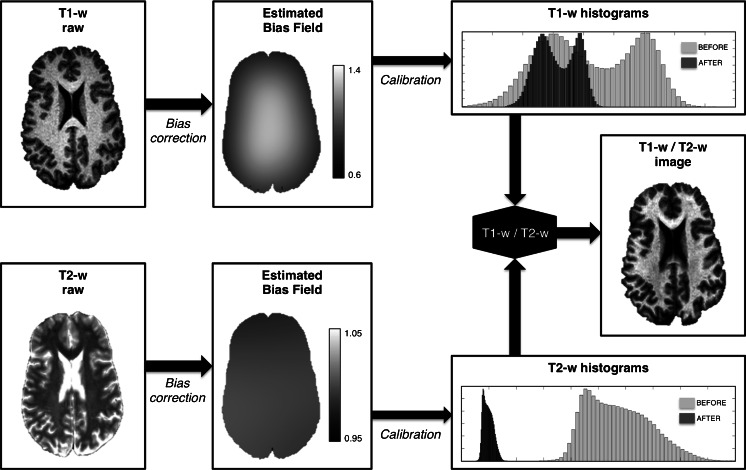
Processing workflow for the generation of the T1-w/T2-w image. The T1-w and T2-w images are subjected to bias correction to remove the slow intensity variations related not only to the MR hardware but also its interaction with the subject’s cranial tissue. Subsequently, the bias-corrected images are calibrated using intensities from masks outside the brain, thereby avoiding problems related to the use of an internal calibration. Finally, the multi-modal T1-w/T2-w image is calculated as the ratio of the standardized T1-w and T2-w images

T1-w and T2-w images were preprocessed and combined using a dedicated workflow as described in [8]. This includes bias correction and intensity calibration on each of the two images and the subsequent calculation of the ratio between preprocessed T1-w and T2-w images (Fig. 1).

**Fig. 2:**
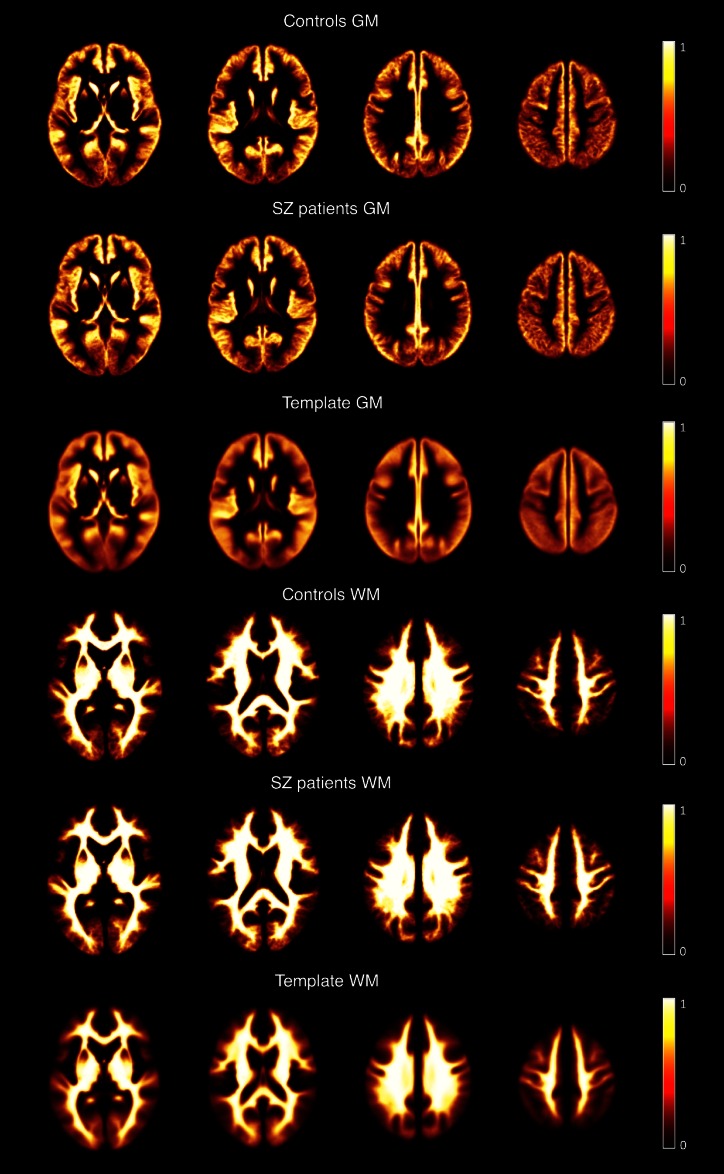
GM and WM maps for the fBIRN dataset images. We tested the registration to MNI space of images from healthy controls and schizophrenic patients. To this end, the gray and white matter masks in subject space were transformed to MNI space by applying the spatial normalization estimated from T1-w and T2-w images. The MNI-space masks belonging to the control and schizophrenia groups were then averaged across subjects to create group-specific probability maps (with values between 0 and 1). The probability maps associated with the MNI template are shown for comparison

The entire processing of the T1-w/T2-w image was conducted using SPM8 (Wellcome Trust Centre for Neuroimaging, London, UK). Initially, the original T2-w image was coregistered to the T1-w image through a rigid-body transformation [10]. Then, the T1-w and T2-w images were separately subjected to bias correction using the “New Segmentation” tool implemented in SPM8 [11, 12]. The input parameters for the bias correction algorithm, namely the smoothing and the regularization parameters, were set at their default value. After correcting for intensity non-uniformity, the T1-w and T2-w images were further processed to standardize their intensity by using a linear scaling procedure systematically described in Ganzetti et al. [8]. Eventually, after calibrating T1-w and T2-w images, their ratio was calculated to generate the calibrated T1-w/T2-w image. To conduct statistical comparisons between groups, we spatially transformed the T1-w/T2-w image in subject space to the Montreal Neurological Institute (MNI) space and carefully checked the effectiveness of this registration (Fig. 2).

### Statistical analysis

First of all, we assessed the T1-w/T2-w technique against other processing strategies and modalities. To this end, we conducted an analysis using small regions of interest (ROIs). Using the MarsBaR toolbox (http://marsbar.sourceforge.net), we created spherical ROIs of 6 mm radius, centered over the coordinates reported in a recent meta-analysis [13] summarizing structural alterations in schizophrenia as revealed by a conspicuous number of medical imaging studies (see Table 1 for details). We also included in this analysis the masks of gray matter (GM), white matter (WM), and cerebrospinal fluid (CSF), as provided by the International Consortium for Brain Mapping (http://www.loni.usc.edu/ICBM). For each ROI, we used an unpaired *t* test to assess the difference between the T1-w/T2-w values in the schizophrenia and control groups. Specifically, we calculated the average intensity across the voxels of a given ROI for *n* controls and *m* schizophrenic patients, to define the vectors *C* and *SZ*, respectively. The *t* score associated with the unpaired *t* test between the two vectors was obtained using the formula:

1 $$ {t}_{\mathrm{ROI}}=\frac{\mu_C-{\mu}_{SZ}}{\sqrt{\frac{s_C^2}{n}+\frac{s_{SZ}^2}{m}}} $$

where *μ*_*C*_ and *μ*_*SZ*_ are the sample means and *s*_*C*_ and *s*_*SZ*_ the sample standard deviations for controls and patients, respectively. Statistical significance was assessed by using permutation-based non-parametric testing [14]. For this analysis, we examined the results of the unpaired *t* test on the following: 1) the ratio between the unprocessed T1-w and T2-w images, 2) the ratio between unbiased, but not calibrated T1-w and T2-w images, 3) the ratio between calibrated, but not unbiased T1-w and T2-w images, 4) unbiased and calibrated T1-w images, and 5) unbiased and calibrated T2-w images. Using *t* values as an indicator of the capability of discriminating between schizophrenic and control groups, we evaluated the effects of bias correction and image calibration individually, as well as the enhancement of contrast introduced by the ratio between T1-w and T2-w images.

**Table 1:** Regions of interest: MNI coordinates

ROI name	MNI coordinates		
	*x*	*y*	*z*
Left insula	−38	12	−3
Right insula	44	10	−4
Left thalamus: medial dorsal nucleus	−3	−13	1
Left frontal lobe: medial frontal gyrus (BA10)	−1	53	−10
Left anterior cingulate (BA32)	−1	50	−12
Left deep frontal lobe	−12	1	−13
Right frontal lobe: medial frontal gyrus (BA9)	3	55	15
Left posterior cingulate (BA23)	−7	−61	15
Right globus pallidus	18	2	−1
Left caudate head	−5	10	−1

Spherical ROIs of 6 mm radius were defined according to the meta-analysis of Ellison-Wright and Bullmore [13]. Coordinates of the sphere center are in MNI stereotaxic space

In a second step, we focused on T1-w/T2-w signal differences between patients and controls. We calculated image histograms and compared them for the two groups, in order to examine whether schizophrenia patients had globally increased/decreased values with respect to age-matched healthy controls. Subsequently, we analyzed T1-w/T2-w values more in detail using an additional ROI analysis. The ROIs were based on masks for specific GM and WM regions. The GM regions included the following: frontal lobe, parietal lobe, occipital lobe, temporal lobe, insula, cerebellum, putamen, caudate, and thalamus. These were defined using the ICBM probabilistic atlas provided by the International Consortium for Brain Mapping (http://www.loni.usc.edu/ICBM). The WM regions included the following: anterior thalamic radiation (ATR), superior longitudinal fasciculus (SLF), inferior longitudinal fasciculus (ILF), inferior fronto-occipital fasciculus (IFOF), pontine crossing tract (PCT), corticospinal tract (CST), forceps major, forceps minor, and middle cerebellar peduncle (MCP). These ROIs were defined using the DTI-based WM atlas of the Laboratory of Brain Anatomical MRI, John Hopkins University School of Medicine, Baltimore, MD, USA (http://cmrm.med.jhmi.edu).

Eventually, we carried out a whole-brain analysis for a more exploratory investigation of regional differences in T1-w/T2-w intensity. In particular, we generated a *t* score map from the T1-w/T2-w data by applying the formula in Eq. 1 to each voxel rather than to an ROI. We used a statistical threshold that was defined using permutation-based non-parametric inference [14], applying threshold-free cluster enhancement (TFCE) to account for multiple comparisons [15]. The resulting probability map (corrected for multiple comparisons) was thresholded at *p* = 0.005, highlighting brain regions with significantly larger differences between the two groups.

## Results

**Fig. 3:**
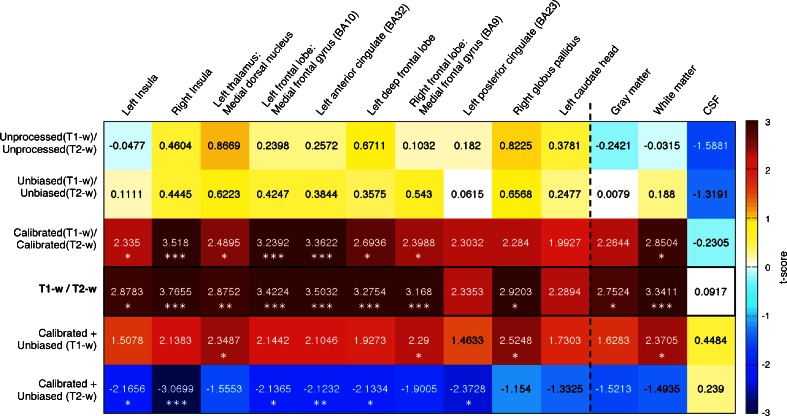
Impact of different processing of T1-w and T2-w images. We conducted an ROI analysis to validate the various stages of the T1-w/T2-w processing protocol. The ROIs were spherical with a 6-mm radius, positioned in MNI space on the basis of coordinates reported by Ellison-Wright and Bullmore (2010). We assessed T1-w/T2-w signal differences between schizophrenic and control groups by using *t* tests. Eventually, we extended the analysis to three additional regions corresponding to the gray matter (GM), white matter (WM), and cerebrospinal fluid (CSF). The figure contains *t* scores resulting from the *t* tests between groups. Areas exhibiting significant group differences, which were assessed by permutation-based non-parametric testing, are marked with *stars* (*p < 0.05; **p < 0.01; ***p < 0.005 uncorrected)

First of all, we used selected ROIs that are considered key regions in the schizophrenia pathology to validate T1-w/T2-w approach and accurately examine its effectiveness for the mapping of differences between patients and healthy individuals (Fig. 3). We tested 10 ROIs in total, of which eight were significant (*p* < 0.05) when using the T1-w/T2-w images. Overall, the results of the unpaired *t* tests comparing the T1-w/T2-w values of the schizophrenia and control groups demonstrated that the bias correction, and more considerably the external calibration, yielded increased T1-w/T2-w signal differences between groups. The T1-w/T2-w workflow, which combines bias correction and image intensity calibration, indeed yielded higher *t* scores than images obtained by using only one or none of these processing steps. Notably, the *t* scores obtained using only the calibration procedure were much closer to T1-w/T2-w *t* scores than those obtained with only the bias correction. We observed a similar trend when using GM and WM as ROIs. A remarkable result was that the difference between patients and controls in the CSF, where no structural differences were expected, was minimal for the T1-w/T2-w approach. We also assessed the importance of taking a multi-modal approach combining T1-w and T2-w images. Accordingly, we compared the T1-w/T2-w results with those obtained using the unbiased and calibrated T1-w and T2-w images independently. In this case as well, we obtained higher *t* scores for the T1-w/T2-w in the schizophrenia-specific regions, as well as in GM and WM. In turn, the *t* score in the CSF was lower for the T1-w/T2-w image than for separate T1-w and T2-w images, suggesting that the ratio minimizes spatial inhomogeneities that may be unrelated to the structural properties of the brain and are present both in T1-w and T2-w images.

**Fig. 4:**
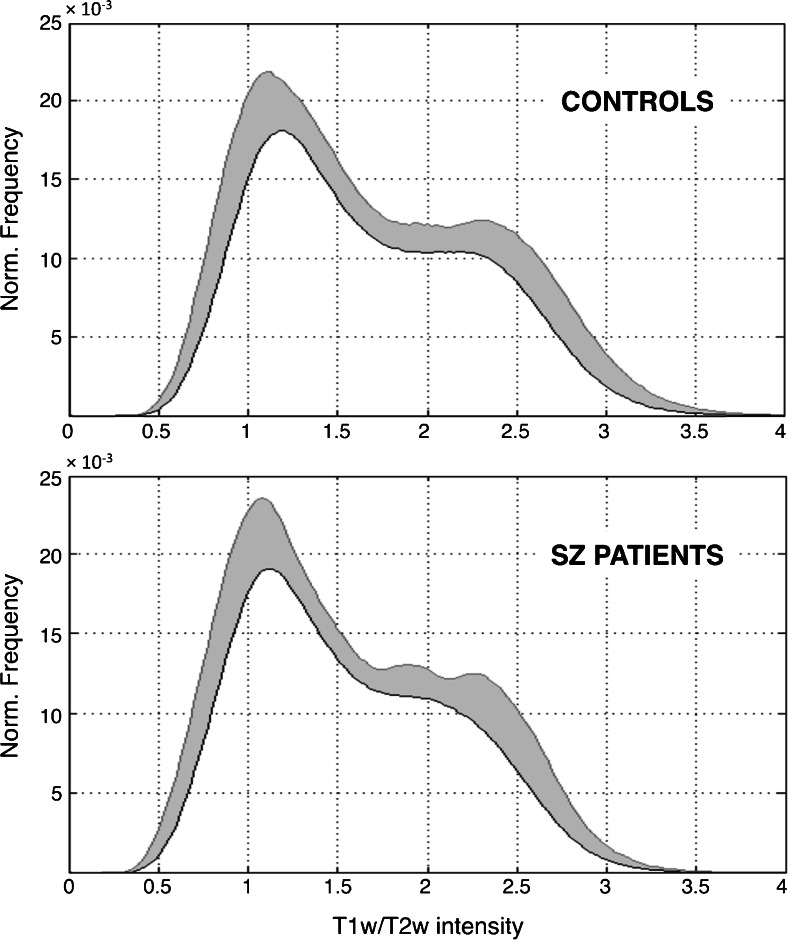
Histograms of T1-w/T2-w intensities in patients and controls. Mean T1-w/T2-w histograms (with standard deviation *shaded*) are shown for patients and controls, separately. Two main peaks are visible in the histograms of both groups. The intensity of the first peak (associated with the gray matter) is similar in patients and controls, whereas the second peak (associated with the white matter) has a lower intensity in patients than in controls. Furthermore, the schizophrenic patients are characterized by a higher standard deviation than healthy subjects

**Fig. 5:**
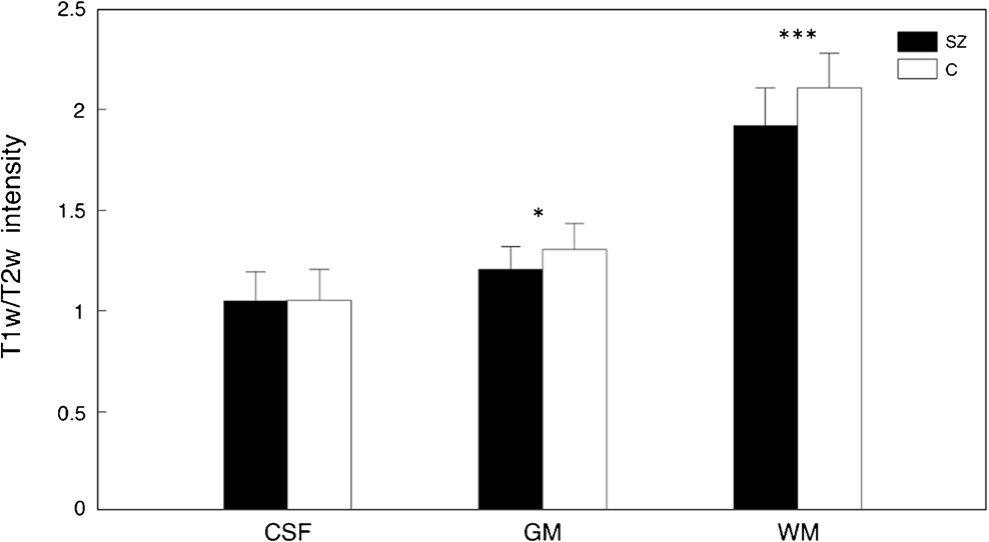
T1-w/T2-w intensities in patients and controls for CSF, GM, and WM. Mean T1-w/T2-w values (with standard deviation) are shown for patients and controls. Both GM and WM have a lower intensity in patients than in controls, with the second yielding the largest difference. Furthermore, no statistical difference was observed for the CSF (*p < 0.05; **p < 0.01; ***p < 0.005 uncorrected)

After this initial validation step, we focused on the calibrated T1-w/T2-w images and examined the global distribution of their intensities in schizophrenic patients and age-matched healthy volunteers, respectively. The patient group revealed globally reduced values, along with increased inter-subject variability (Fig. 4). More specifically, the reduction appeared more marked in regions with higher T1-w/T2-w intensity, which can be putatively attributed to the WM. To confirm this result, we analyzed average T1-w/T2-w intensities within GM, WM, and CSF (Fig. 5). Accordingly, we found significant T1-w/T2-w reductions in schizophrenia for GM and WM, with the latter yielding the largest difference compared to controls.

**Fig. 6:**
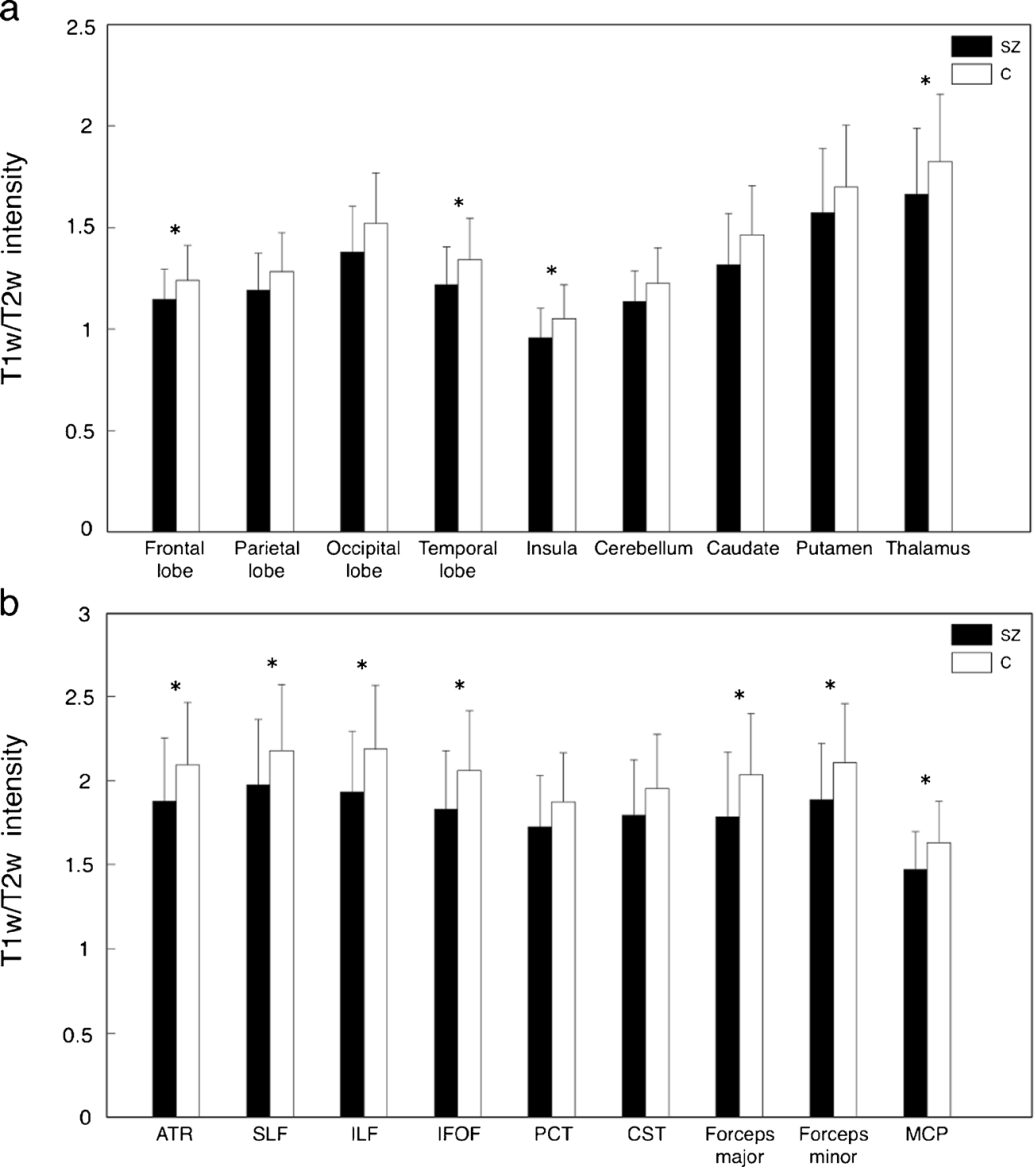
T1-w/T2-w intensities in patients and controls: GM and WM regions. Mean T1-w/T2-w values (with standard deviation) are shown for patients and controls. A global reduction of GM (**a**) and WM (**b**) intensity was reported. In terms of GM structures, the temporal lobe showed the largest significant reduction. Among the subcortical nuclei, caudate and thalamus exhibited decreased values. No statistical difference was revealed in the parietal lobe and putamen. As for WM tracts, the left hemisphere along with the frontal region showed the largest reduction (*p < 0.05; **p < 0.01; ***p < 0.005 uncorrected)

We then focused our investigation on specific brain regions within the GM and WM for a local assessment of image intensities (Fig. 6). No regions with T1-w/T2-w increase were found in schizophrenic patients. Among the GM regions, the frontal lobe, temporal lobe, and insula showed significant reduction (*p* < 0.05). The parietal lobe, occipital lobe, and cerebellum did not show a significant difference. As for the subcortical nuclei, only the thalamus showed a significant reduction (*p* < 0.05), whereas no differences were found in putamen and caudate nucleus. Among the WM regions included in the analysis, the ATR, SLF, ILF, IFOF, forceps major, forceps minor, and MCP showed a significant reduction (*p* < 0.05) in T1-w/T2-w values, whereas no significant difference was detected in PCT and CST.

**Fig. 7:**
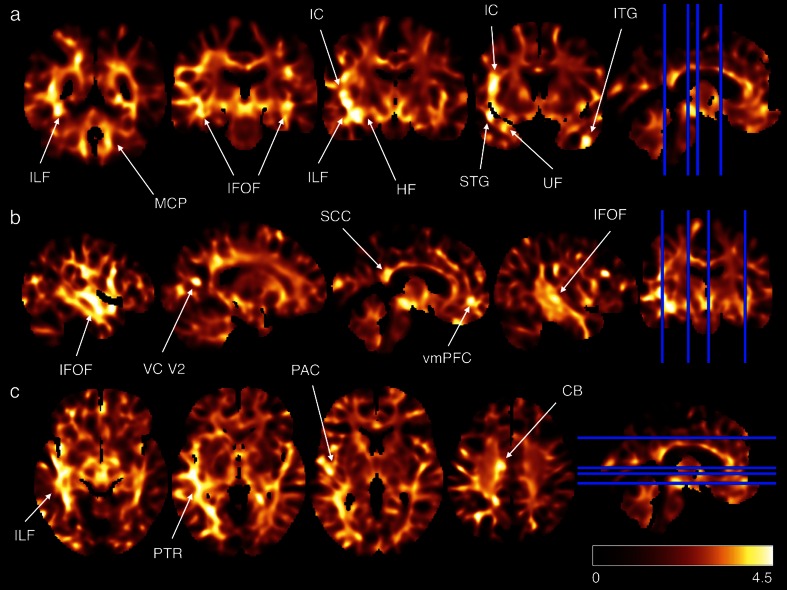
T1-w/T2-w differences between schizophrenia patients and healthy controls: exploratory analysis. T1-w/T2-w differences in schizophrenic subjects compared with age-matched healthy controls were assessed on a voxel-wise basis using an unpaired *t* test. Regions with significantly greater T1-w/T2-w values in controls are shown in *yellow*-*white*. No regions were found with significantly greater T1-w/T2-w values in patients. The maps were represented using coronal (**a**), sagittal (**b**), and axial (**c**) sections. Fundamental structures with high *t* scores are indicated using *arrows*: *ILF* inferior longitudinal fasciculus, *MCP* middle cerebellar peduncle, *IFOF* inferior fronto-occipital fasciculus, *IC* insular cortex, *HF* hippocampal formation, *UF* uncinate fasciculus, *STG* superior temporal gyrus, *ITG* inferior temporal gyrus, *VC V2* visual cortex V2, *vmPFC* ventromedial prefrontal cortex, *PTR* posterior thalamic radiations, *PAC* primary auditory cortex, *SCC* splenium of corpus callosum, *CB* callosal body

**Fig. 8:**
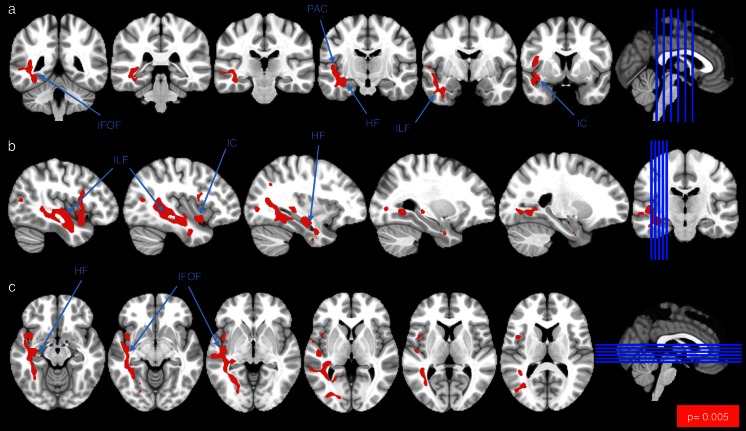
T1-w/T2-w differences between schizophrenia patients and healthy controls: thresholded map. T1-w/T2-w differences in schizophrenic subjects compared with age-matched healthy controls were assessed on a voxel-wise basis using an unpaired *t* test. Regions with significantly reduced T1-w/T2-w values in patients (*p* < 0.005, TFCE corrected) are shown in *red*. No regions were found with significantly greater T1-w/T2-w values in patients. The maps were overlaid on a standard MNI template and represented using coronal (**a**), sagittal (**b**), and axial (**c**) sections. Selected structures with significant *t* scores are indicated using *arrows*: *PAC* primary auditory cortex, *HF* hippocampal formation, *IC* insular cortex, *ILF* inferior longitudinal fasciculus, *IFOF* inferior fronto-occipital fasciculus

To corroborate and extend our T1-w/T2-w results beyond the selected ROIs, we repeated the same analysis at the single-voxel level, thereby generating an unthresholded map of differences between groups (Fig. 7). Schizophrenic patients exhibited hypointensity in areas thought to play a central role in the pathology. In terms of GM structures, we found T1-w/T2-w reductions in correspondence of the left hippocampal formation (HF), right inferior temporal gyrus (ITG), left superior temporal gyrus (STG), left visual cortex (VC) V2, right ventromedial prefrontal cortex (vmPFC), left primary auditory cortex (PAC), and bilateral insular cortex (IC). As for WM tracts, decreased values were reported in the MCP, ILF, IFOF, uncinate fasciculus (UF), posterior thalamic radiations (PTR), splenium of corpus callosum (SCC), and callosal body (CB). The differences between schizophrenia and control groups were particularly significant (*p* < 0.005, TFCE corrected) in the left temporal regions and specifically in PAC, HF, and IC for the GM and in ILF and IFOF for the WM (Fig. 8).

## Discussion

The T1-w/T2-w approach was initially proposed by Glasser and Van Essen [9] for a qualitative mapping of the myelin present in the gray matter of an individual subject. It was subsequently further developed by Ganzetti et al. [8] for quantitative assessment of myelin content in the whole brain. In this current study, we evaluated the potential utility of the T1-w/T2-w approach by conducting an analysis on T1-w and T2-w data collected in schizophrenic patients and age-matched controls. A considerable number of studies have focused on the etiology behind structural alterations in schizophrenic patients, though no consensus has yet been fully reached. As for the GM, detailed examinations of tissue reductions in the schizophrenic brain have been commonly achieved by voxel-based morphometry (VBM) [13], while diffusion tensor imaging (DTI) has attracted particular attention for the mapping of reduced anisotropic patterns within WM tracts possibly linked to myelination changes [16, 17]. Nonetheless, cortical atrophy [18–20], neuroinflamation [21], degradation of the myelin sheath [22, 23], and accumulation of extra-cellular water [24] have most frequently been related to the disease. All these factors can, in principle, be detected as T1-w/T2w intensity differences between patients and healthy individuals.

We began our investigations by selecting a set of brain regions that are most robustly found to be impaired in schizophrenia patients, based on the recent meta-analysis of Ellison-Wright and Bullmore [13]. The results of our ROI analysis showed that T1-w/T2-w image had increased T1-w/T2-w signal differences between groups compared to images obtained with either bias correction or linear calibration. Furthermore, our analysis also suggested that the ratio between the bias-corrected and calibrated T1-w and T2-w images yielded larger detection accuracy with respect to the single modalities taken separately. Not only was the ratio between T1-w and T2-w critical for detecting true positives but also allowed for an attenuation of any putatively detrimental component shared between the two modalities. As shown by the CSF results, subsequent pipeline stages generate smaller difference when comparing schizophrenic patients and healthy controls, whereas in those regions characterized by a high probability of finding structural alterations, the difference becomes significant. Furthermore, the results of the analysis conducted using WM and GM as ROIs (Fig. 5) suggested a global reduction of T1-w/T2-w intensity, with the first one showing relatively more reduced values in schizophrenia. This is in line with several functional and structural studies suggesting that psychotic and cognitive symptoms are linked to abnormalities of cortical tissues, such as in limbic and frontal-temporal networks [13, 25–29]. Likewise, the presence of differences in WM implies fiber tract impairment as a major key feature in the pathology [30, 31]. Thus, functional dysfunctions in brain networks may result from structural changes both in WM and in GM [32, 33]. These results are in line with current studies suggesting that global disruptions in WM connectivity combined with local GM alterations may be the foundation for the clinical and cognitive outcome of the pathology [17, 22, 23].

In terms of local structural impairments in GM (Figs. 6, 7), we found an agreement between our findings and those of voxel-based morphometric studies, in particular for the insula, thalamus, HF, and temporal and frontal lobes [13, 28, 34–36]. The results of the ROI and voxel-wise analyses were in large agreement, with the voxel-wise analysis revealing additional areas such as HF and PAC (Fig. 8). Our analyses revealed prominent alterations in schizophrenic patients to be localized in the left hemisphere, in correspondence of the PAC [28, 37–40] (Fig. 8). Weakened activity in this brain region was also reported by Gaser and coworkers [41], who suggested that abnormalities in this area may underlie hallucination-specific disorders [42]. Regarding subcortical structures, no significant difference was reported in the putamen and caudate (Fig. 6). However, we found a significant T1-w/T2-w reduction in the right globus pallidus (Fig. 3). Several structural studies showed alterations in the basal ganglia, among which the globus pallidus, to underlie problems in higher cognitive functions such as attention and working memory, which are typically impaired in schizophrenia [43]. Not only are our findings consistent with previous neuroimaging studies but also with the main behavioral features of the pathology. For instance, we found significant differences in the frontal cortex, in particular the vmPFC, which has been implied in social cognition and especially in emotion regulation [44, 45]. Also, alterations found in the insula are consistent with problems related to mental representation of emotions [46].

As for the WM results, our analyses identified two major locations of T1-w/T2-w reduction in schizophrenic brains: one in the frontal areas and the other in the left temporal region (Figs. 6, 7). Similar results have also been reported in several meta-analyses, showing consistent WM aberrations in prefrontal area pathways [16, 17, 23]. Both our ROI- and voxel-based results highlighted significantly reduced values in the left temporal lobe compared to the contralateral (Fig. 8), with the ILF and the temporal radiations of IFOF as the most affected in patients. Reductions of WM in patients were also reported in the cerebellum; in particular, we observed a significant reduction in the middle cerebellar peduncle (MCP) (Fig. 6b). These findings are consistent with previous structural and functional neuroimaging studies linking cerebellar deficits to neurological signs in schizophrenia, via impaired coordination of mental processes [47, 48]. Smaller cerebellar volumes for both hemispheres [49] along with WM volume reductions in the cerebellum [17, 50] are often reported. Furthermore, the observed impairments in the ATR (Fig. 6b), which contains fibers interconnecting the thalamus and prefrontal cortex, are consistent with the results of Mamah et al. [51], who documented that ATR integrity is inversely correlated with cognitive dysfunctions. In conclusion, our study mostly confirmed and extended the “disconnection syndrome” theory in the schizophrenic brain [17, 48, 52].

More generally, our current results from schizophrenia data suggest that the T1-w/T2-w technique can be more reliably used to map differences compared to healthy controls than methods based either on T1-w and T2-w images. A number of potential limitations of our study should be however considered. The first one is that we used only a limited amount of datasets for the method validation. Since images collected with very different pulse sequences may generate inconsistent results in terms of image contrast, we suggest the T1-w/T2-w pipeline should be used on a broader range of datasets to evaluate its potential use for large-scale neuroimaging analyses. Further investigations are warranted to confirm our preliminary findings and to explore the utility of the method on different brain pathologies. In line of principle, better spatial accuracy can be achieved with images acquired at high spatial resolution. Our results suggested that image resolution is not critical for the use of the T1-w/T2-w technique. The availability of T1-w and T2-w images at high spatial resolution is critical for the use of surface-based mapping methods. This would provide more accurate spatial registration across subjects as well as would permit evaluating cortical folding patterns and cortical thickness together with T1-w/T2-w image intensity. The integration of surface-based methods in the pipeline for the T1-w/T2-w analysis may be an important methodological advancement.

## Conclusion

In summary, our findings suggest that the T1-w/T2-w technique can be reliably used to map differences in brain structure between patients and healthy individuals with a greater accuracy than by using methods based on T1-w or T2-w images alone, supporting the definition of more reliable disease biomarkers. The use of other MR-based techniques (e.g., proton spectroscopy, magnetization transfer techniques, and relaxation times) and positron emission tomography (PET) can certainly help to clarify the pathological processes associated with the detected T1-w/T2-w signal differences between patients and healthy individuals.
